# Enhanced anti-glioma efficacy of biodegradable periodic mesoporous organosilica nanoparticles through target delivery of chemotherapeutics

**DOI:** 10.1007/s10856-023-06747-x

**Published:** 2023-10-04

**Authors:** Min Dong, Ying Liu, Biao Liu, Jin Peng, Yuxia Tang, Guangming Lu, Haibin Shi, Feipeng Zhu

**Affiliations:** 1 Department of Comparative Medicine, Jinling Hospital, School of Medicine, Nanjing University, 305 East Zhongshan Road, Nanjing, 210002 PR China; 2 School of Intelligent Manufacturing and Electronic Engineering, Wenzhou University of Technology, Wenzhou, 325025 PR China; 3 Intervention Department, Chenggong Hospital Affiliated to Xiamen University, 94-96 Wenyuan Road, Xiamen, 361003 PR China; 4 Department of Radiology, The First Affiliated Hospital of Nanjing Medical University, 300 Guangzhou Road, Nanjing, 210029 PR China; 5 Department of Medical Imaging, Jinling Hospital, School of Medicine, Nanjing University, 305 East Zhongshan Road, Nanjing, 210002 PR China; 6 Department of Interventional Radiology, The First Affiliated Hospital of Nanjing Medical University, 300 Guangzhou Road, Nanjing, 210029 PR China

## Abstract

**Abstract:**

Glioma is the most common malignant tumor of the brain and enhancing the efficacy of chemotherapy in glioma is critical for improving patients’ prognosis. In this study, a glioma-targeting drug delivery system is constructed using biodegradable periodic mesoporous organosilica nanoparticles (PMO) that are modified with lactoferrin (Lf) ligands. The obtained PMO is doped with thioether groups and can be degraded in the high concentration of glutathione in tumor cells. The surface area and pore volume of PMO are 772 cm^2^/g and 0.98 cm^3^/g, respectively and the loading capacity of doxorubicin (Dox) is as high as 20%. The results of the confocal laser scanning microscope show that the uptake of PMO-Lf@Dox by C6 cells is higher than PMO@Dox. The quantitative analysis of the flow cytometer further demonstrates that more PMO-Lf@Dox enter C6 cells, indicating that the modification of lactoferrin can significantly increase the uptake of C6 cells. Finally, the therapeutic efficacy results show that Lf-modified PMO enhances the inhibitory effect of Dox on C6 cells when incubated for 24 h and 72 h. In summary, this lactoferrin receptor-mediated PMO drug carrier with biodegradability in glutathione in tumor cells can be used to enhance drug delivery into glioma without long-term accumulation in vivo.

**Graphical abstract:**

**Figure:**
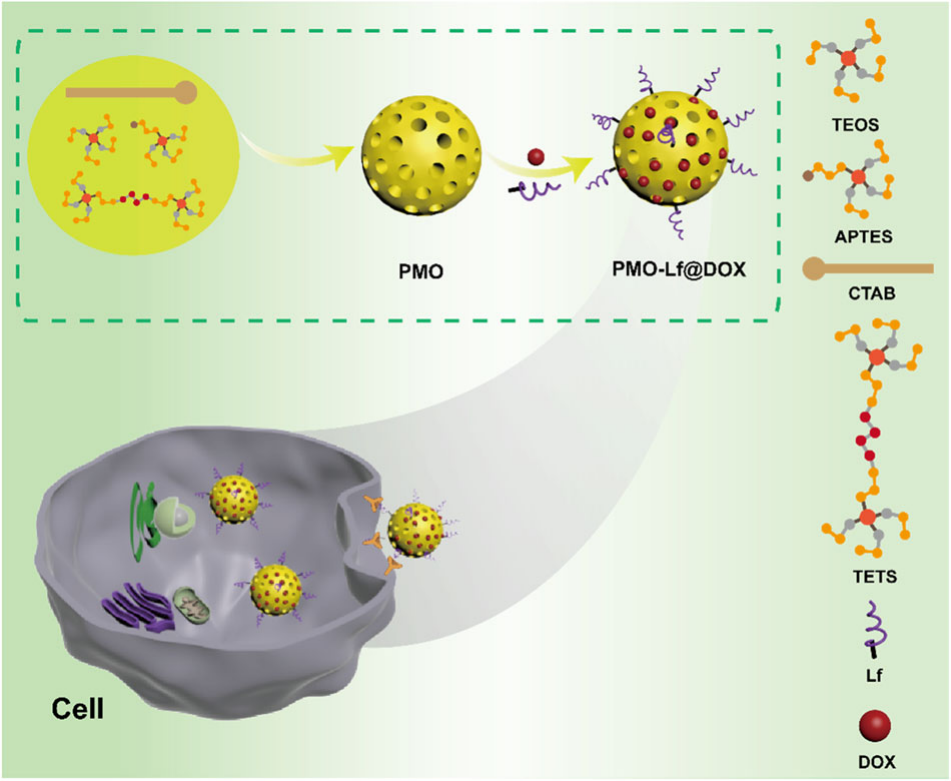
In this study, a glioma-targeting drug delivery system is constructed using periodic mesoporous organosilica nanoparticles (PMO) that modified with lactoferrin (Lf) ligands. This lactoferrin receptor-mediated PMO drug carrier can be used to enhance drug delivery into brain glioma.

## Introduction

Glioma is the most common primary malignant cancer in the brain with high infiltration and the prognosis is very poor [1]. Chemotherapy is still one of the main treatment methods, but the delivery and effective accumulation of drugs in glioma sites are very difficult [2, 3]. Nano-drug delivery system is an effective method to improve the efficiency of drug delivery in glioma by surface-modification of the glioma-targeting receptors [4].

Currently, glioma-targeting ligands include proteins, peptides, aptamers, small molecules, and so on [5, 6]. Among them, lactoferrin (Lf) is one of the most commonly used ligands due to the high expression of its receptor and high affinity in glioma [7–9]. It also has the advantage of low expression in the healthy tissues, thereby no competition with exogenous ligands [10, 11]. Bin et al. [12]. evaluated the glioma targeting efficiency of three different ligands and found that Lf can significantly enhance tumor cellular uptake. Pang, et al. [13] showed that Lf-conjugated polymersomes significantly improve the drug delivery efficiency. After decorating silica nanoparticles with Lf, Janjua and colleagues [14] found that USLP-NH2-Cy5-PEG-LF not only shown improved permeability through the blood-brain barrier, but also exhibited increased induction of glioblastoma cell apoptosis in U87 and GL261 cell lines compared with pure Temozolomide in vitro. Qi et al. [15] found that liposomes modified with integrin αvβ3 and Lf exhibited enhanced targeting of U87-MG cells showed that Lf-conjugated polymersome can significantly improve the drug delivery efficiency.

However, the previous research on Lf mainly focuses on assembling Lf itself into nanoparticles or compounding it with liposomes and polymers to mediate drug delivery. They have certain limitations, including complex synthesis steps and limited stability in the circulatory systems [14–16]. Periodic mesoporous organosilica nanoparticles (PMO) with the characteristics of high specific surface area, large pore volume, and various organic groups for easy modification, have been widely used for drug delivery [17–19]. Traditional PMO may be difficult to degrade in vivo, and the problem of long-term accumulation in vivo has attracted researchers’ attention, so biodegradable PMO can overcome this challenge. Up to date, it has not been reported for drug delivery by using lactoferrin-modified degradable PMO nanoparticles.

**Scheme 1:**
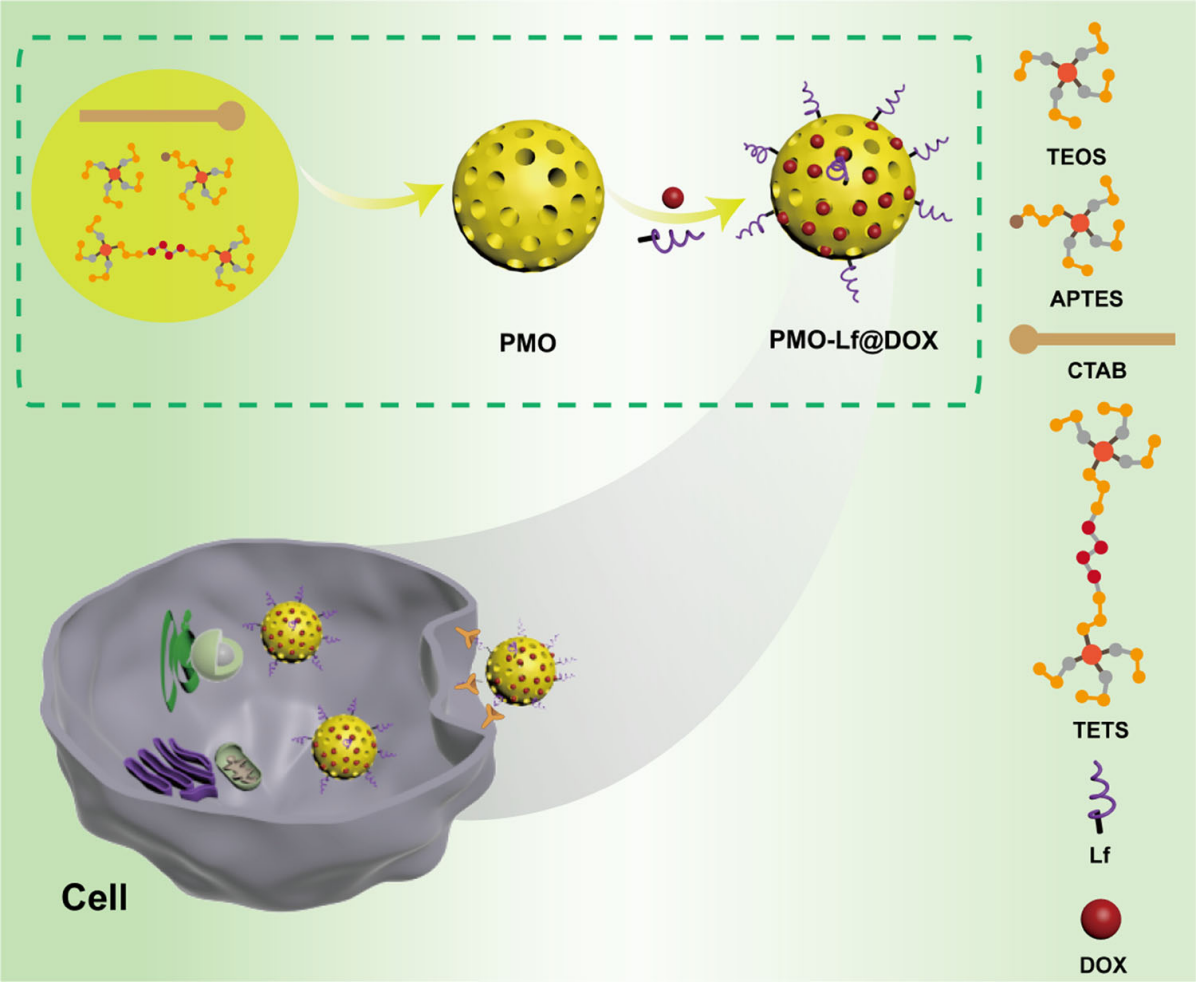
Schematic of the biodegradable periodic mesoporous organosilica nanoparticles (PMO) as effective anticancer platforms. A glioma-targeted drug delivery system is constructed using thioether groups doped PMO that modified with lactoferrin ligands (PMO-Lf) and loaded with doxorubicin (PMO-Lf@Dox). This lactoferrin receptor-mediated PMO drug carrier can be degraded by the high concentration of glutathione (GSH) in tumor cells and enhance drug delivery into glioma by specific receptor-mediated cellular uptake. Tetraethyl orthosilicate (TEOS), 3-aminopropyltriethoxysilane (APTES), cetyltrimethylammonium bromide (CTAB), Bis[3-(triethoxysilyl) propyl] tetrasulfide (TETS)

Herein, we designed biodegradable PMO nanoparticles doped with thioether groups, which can be broken by the high concentration of glutathione (GSH) in tumor cells. Then glioma-targeting drug delivery system was constructed based on lactoferrin ligands modification on the surface of PMO (PMO-Lf). PMO-Lf was loaded with chemotherapy drug doxorubicin (PMO-Lf@Dox) to improve the delivery efficiency of Dox in glioma cells (Scheme 1). Since rat glioma cells C6 express Lf receptor [20], this study tested the delivery efficiency in C6 cells. This receptor-mediated PMO drug carrier can be used to enhance drug delivery into glioma and improve the chemotherapeutic efficacy of glioma.

## Materials and methods

### Materials

Anhydrous ethanol, concentrated ammonia aqueous solution (NH_3_•H_2_O, 25 wt%), tetraethyl orthosilicate (TEOS), Bis[3-(triethoxysilyl) propyl] tetrasulfide (TETS), dimethyl sulfoxide (DMSO), cetyltrimethylammonium bromide (CTAB), concentrated hydrochloric acid (HCl, 37%) were purchased from Sinopharm Chemical Reagent Co., Ltd. (China). 3-aminopropyltriethoxysilane (APTES) was purchased from Sigma-Aldrich (USA). Cell culture medium (Dulbecco’s Modified Eagle’s Medium, DMEM), Dimethyl sulfoxide (DMSO), penicillin, fetal bovine serum (FBS) and Trypsin were provided by Gibco (USA). Bovine serum albumin (BSA) was provided by Promag (USA). 3-(4,5-dimethylthiazol-2)-2,5-diphenyltetrazolium bromide (MTT) and lactoferrin (Lf) was purchased from Nanjing Kengen (China). Doxorubicin hydrochloride (Dox) purchased from Sangon (China). All water was deionized water (Millipore, USA) with a resistivity of 18.2 MΩ/cm. Human embryonic kidney cells 293T, rat glioma cells C6 were purchased from American Type Culture Collection (ATCC) Cell bank.

### Characterization

Transmission electron microscopy photos are obtained with a microscopy (TEM, Hitachi HT7700, Japan). Fourier transform infrared spectroscopy was detected by using Nicolet Instruments (Nicolet Nexus 870, USA). The particle size and Zeta potential were measured by a ZetaPALS analyzer (Brookhaven, USA).

### Synthesis of PMO

PMO was synthesized according to the literature [21, 22]. Briefly, 0.16 g CTAB was dissolved in a mixture containing 100 mL water and 30 mL ethanol, and then 1 mL ammonia aqueous solution (25%) was added, stirring at 1100 rpm and 35 °C for 1 h. Then 0.25 mL TEOS and 0.25 mL TETS, pipetted for 40 times, was added to the mixture dropwise and reacted for 24 h. The solution was centrifuged at 13,500 rpm for 30 min and washed three times using ethanol. Then the obtained product was dispersed in 120 mL of ethanol and 240 μL of concentrated hydrochloric acid at 60 °C, stirring at 500 rpm for 3 h and repeated for three times to remove CTAB. The obtained product was centrifuged and dispersed in 30 mL of ethanol at 35 °C followed by adding 5 μL of APTES, stirring at 1100 rpm for 1 h, then centrifuged at 13,500 rpm for 30 min, washed with ethanol for three times. The obtained PMO was dispersed in 30 mL ethanol for further use.

### Dox loading and the modification with Lf

For Dox loading, PMO and Dox were mixed in 1 mL deionized water at a weigh ratio of 1: 1, stirred overnight under dark, and then centrifuged and washed three times using water. The supernatant was collected and measured at 488 nm by a UV–Vis spectrophotometer. The obtained product was denoted as PMO@Dox. The loading capacity was calculated according to the following formula: loading capacity (%) = 100×Entrapped Dox/(PMO weight + Entrapped Dox). To modify with Lf, 1 mL of PMO@Dox was mixed with 5 μL of glutaraldehyde (0.025%) at 37 °C for 2 h, followed by centrifugation and washing using water for three times. Then 10 μL of Lf (0.5 mg/mL) was added and shaken overnight.

### Hemolysis experiment

Briefly, 1 mL of blood was washed with saline, centrifuged at 2000 rpm for 5 min and repeated several times until the supernatant is colorless. The obtained red blood cells (RBC) were diluted with 5 mL of saline. Then 0.2 mL of diluted RBCs was mixed with 0.8 mL of PMO with different concentrations and incubated at 37 °C for 2 h. Meanwhile, 0.8 mL of saline and water mixed with 0.2 mL of RBC was negative and positive control, respectively. Finally, all samples were centrifuged at 2000 rpm for 5 min, and the absorbance at 490 nm of the supernatant was measured. Hemolysis rate (%) = 100*(the absorbance of the sample − the absorbance of negative control)/(the absorbance of positive control − the absorbance of negative control).

### In vivo biocompatibility

Animal experiments were conducted in accordance with the animal ethics management regulations of the Jinling Hospital. Male mice were randomly divided into 2 groups for 6–8 weeks, and 3 mice in each group. PMO (60 mg/kg) was injected into the tail vein. One week later, the mice were sacrificed, and the main organs were taken, including the heart, liver, spleen, lung and kidney, and formalin. After fixation, sectioning and HE staining were performed. The other group of mice was injected with saline through the tail vein as a control group.

### In vitro biodegradability

Thioether groups incorporated PMO was dispersed in Glutathione (10 mM) solution for 3 d and 14 d. PMO dispersed in water for 14 d was set as control. The morphology of PMO in different time was observed under TEM.

### Cellular uptake

C6 cells were cultured in DMEM medium containing 10% fetal bovine serum, 100 U/mL penicillin, and 100 ug/mL streptomycin, and incubated in a 37 °C, 5% CO_2_ incubator. Confocal laser scanning microscope (CLSM) and flow cytometer were used to observe and analyze the uptake of PMO@Dox and PMO-Lf@PMO by C6 cells. For CLSM, C6 cells were seeded into 12-well plates, containing a cell slide in each well, at a density of 10^5^ cells per well. After the cells adhere to the well, Free Dox, PMO@Dox and PMO-Lf@PMO at the equivalent concentration of Dox of 5 μg/mL were added to the wells. Then cells were cultured in for 2, 12 or 24 h, washed with PBS, stained with DAPI, and observed under a CLSM. For flow cytometer analysis, the same protocol was performed except that there was not a slide in each well. After incubation for 2, 12 or 24 h, cells were collected after digestion with trypsin. Each group has three replicate wells, and the cells without any treatment were control.

### Cell viability

To investigate the cytotoxicity of PMO, 293T cells were seeded in two 96-well plates at a density of 8.0 × 10^3^/well. After cultured for 24 h, 10, 20, 50, 100, 200 μg/mL of PMO was added into the wells, respectively. For the therapeutic effect of PMO@Dox and PMO-Lf@Dox, C6 cells were seeded in 96-well plates at a density of 8.0 × 10^3^/well. After cultured for 24 h, 1, 2, 5, 8, 10 μg/mL of Dox equivalent concentration was added to each well. After incubation for 24 h or 72 h, MTT assays were used to detect the cell viability. Cells without any treatment were set as control and 5 replicate wells were set for each concentration. The cell survival rate (%) = 100* (the absorbance value of the cell wells of the experimental group/the absorbance value of the cell wells of the blank control group). The experiment was repeated three times.

## Results

**Fig. 1:**
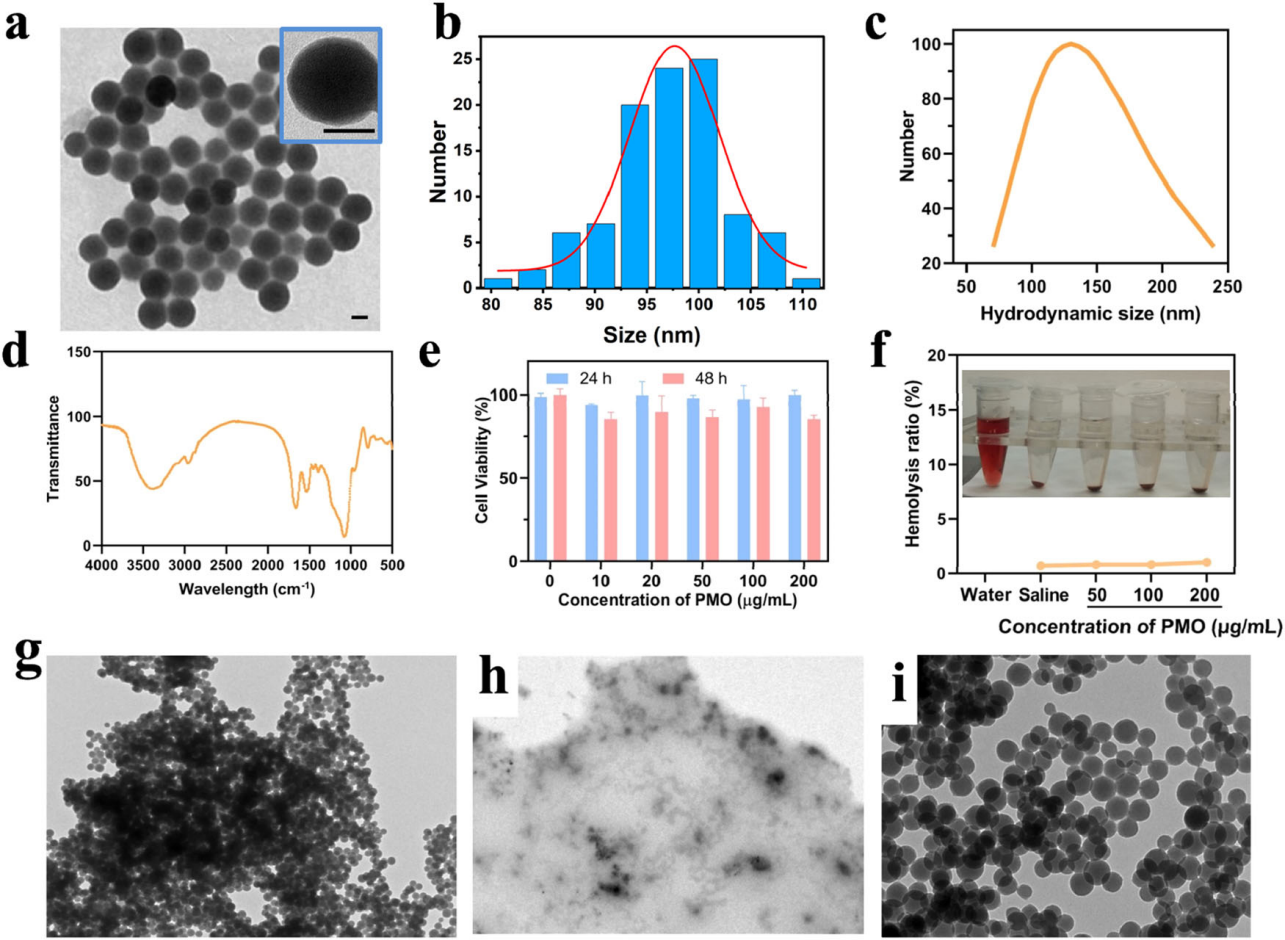
**a** TEM image of PMO, the inset is a high-magnification image. The scale is 50 nm. **b** The size distribution of the prepared PMO, by measuring 80 nanoparticles from the TEM image. **c** The hydrodynamic size of PMO. **d** Fourier transform infrared spectrum of PMO. **e** The cell survival rate of 293T cells incubated with PMO at different concentrations for 24 and 48 h. **f** The ratio of hemolysis after incubating PMO with RBCs and the inset is a photo of hemolysis. RBCs incubated with water and saline are as positive and negative control, respectively. TEM images of disulfide bonds incorporated PMO in Glutathione (10 mM) solution for (**g**) 3 d and (**h**) 14 d. **i** PMO dispersed in water for 14 d was set as control. The scale is 1 μm

**Fig. 2:**
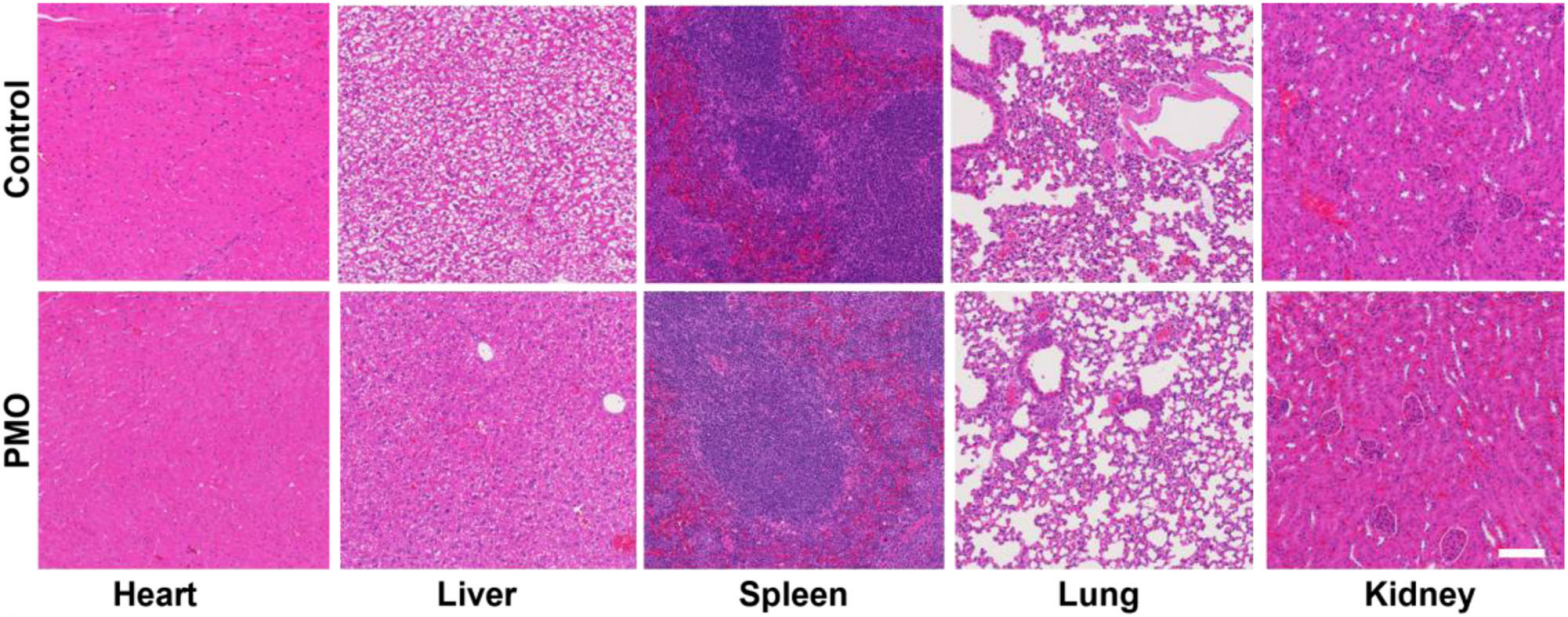
The H&E staining images of the main organs of mice 7 days after the tail vein injection of PMO, the scale is 100 μm

TEM image shows that the obtained PMO is a spherical structure with a size of about 99 nm (Fig. 1a, b). The results of dynamic light scattering indicate that the hydrodynamic size of the PMO is 134 nm, and its polydispersity index is 0.1, indicating that PMO has a good dispersibility in water (Fig. 1c). Fourier transform infrared spectroscopy shows that PMO has a peak at 691 cm^−1^, which is due to the vibration of the C–S bond (Fig. 1d) [22–24]. The cytocompatibility of PMO is assessed by incubating PMO with 293T cells for 24 h and 48 h. It can be found that the cell survival rate is still maintained at 90% when the PMO concentration is as high as 200 μg/mL (Fig. 1e). The results of hemolysis experiments also showed that when the PMO concentration is as high as 200 μg/mL, the red blood cells remained intact, and the hemolysis rate is less than 2% (Fig. 1f). Then we investigated the degradability of thioether groups incorporated PMO in Glutathione (10 mM) solution for 3 d and 14 d and the results show that after 3 d of incubation in GSH, the edges of the PMO nanoparticles became blurred (Fig. 1g), and the PMO nanoparticles were almost completely degraded and no spherical morphology is observed while extending to 14 days (Fig. 1h). In comparison, the morphology of PMO nanoparticles dispersed in water for 14 d remained intact (Fig. 1i). The toxicity of PMO is further evaluated in mice, and it is found that one week after tail vein injection of 60 mg/kg PMO, no obvious changes such as heart, liver, spleen, lung and kidney are seen (Fig. 2). The quality of the different organs in a quantitative approach and the results are shown in Supplementary Table S1. The animal experiments and the weight of the mice was recorded. The results indicated that the material had high biological safety (Supplementary Fig. S1).

**Fig. 3:**
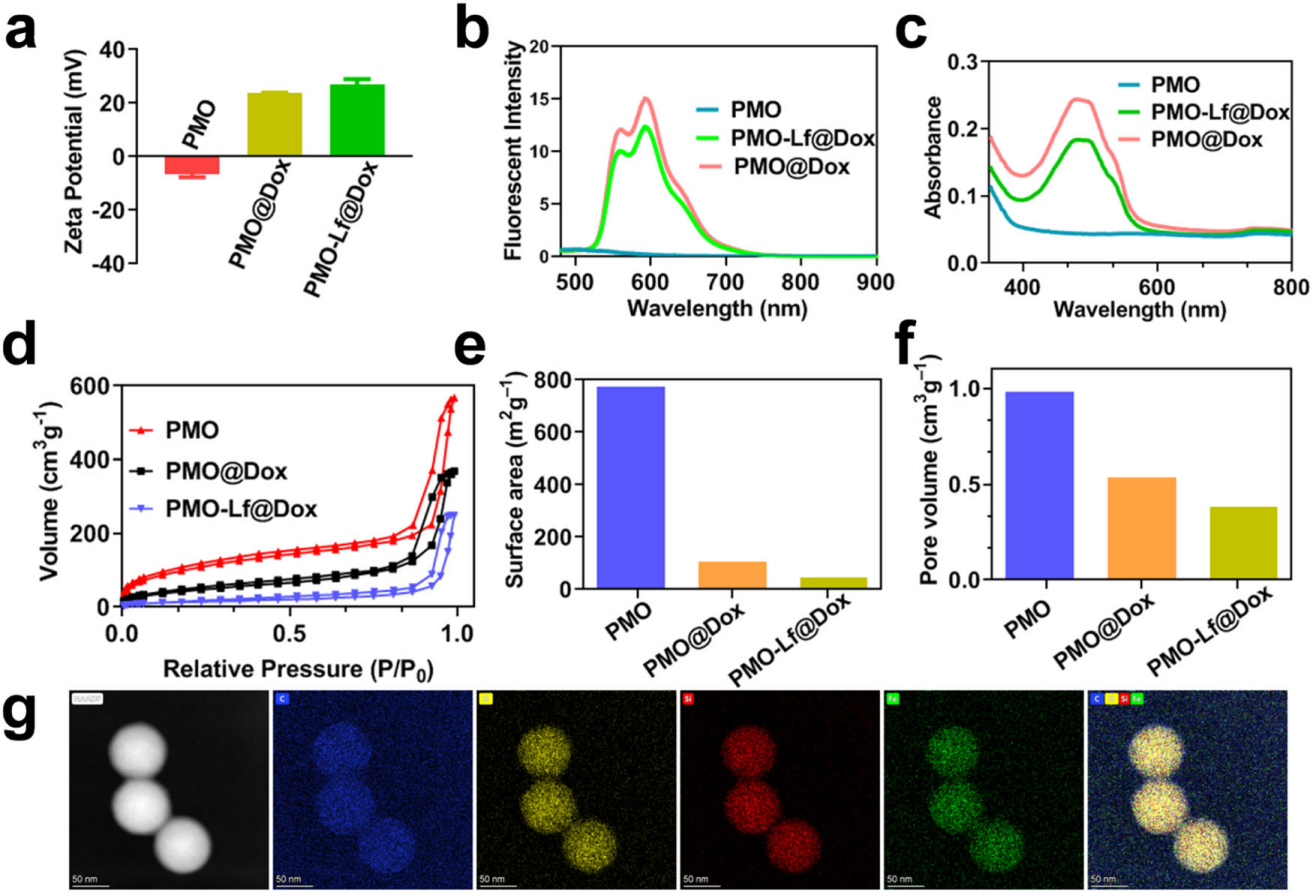
**a** The zeta potential of PMO, PMO@Dox and PMO-Lf@Dox in water. **b** Fluorescence spectrum, **c** UV–Vis absorption spectrum, **d** Nitrogen adsorption and desorption isotherm and **e** the changes of surface areas and **f** pore volumes of PMO, PMO@Dox and PMO-Lf@Dox. **g** Bright field TEM images of PMO-Lf@Dox and X-ray energy spectrum of C element, O element, Si element, and Fe element, and the merge of the four elements

Then the positively charged Dox was loaded to PMO by electrostatic adsorption and the zeta potential rises from −6 mV to 24 mV (Fig. 3a). The characteristic peaks of Dox appear in the UV–Vis spectrum and fluorescence spectrum (Fig. 3b, c), indicating that Dox is successfully loaded. After activated the amino groups on the PMO@Dox surface by using glutaraldehyde, Lf is added and the zeta potential becomes 27 mV, indicating that Lf has been successfully modified. The nitrogen adsorption and desorption isotherm of PMO shows a typical type II curve (Fig. 3d). When the relative pressure is 0.7–1.0, there is an obvious hysteresis loop, indicating the existence of a large hole in PMO. The surface area and pore volume of PMO are as high as 772 cm^2^/g and 0.98 cm^3^/g, respectively. After Dox loaded and Lf modification, the surface area drops to 106 cm^2^/g and 46 cm^2^/g (Fig. 3e), and the corresponding pore volume drops to 0.54 cm^3^/g, and 0.38 cm^3^/g (Fig. 3f), respectively. Quantitative analysis of the amount of Dox in the supernatant and washing solution, the results show that the loading capacity of Dox in PMO is as high as 20%. The element mapping result showed the uniform distribution of the C, Si, O and Fe elements in the framework of PMO-Lf@Dox. (Fig. 3g).

**Fig. 4:**
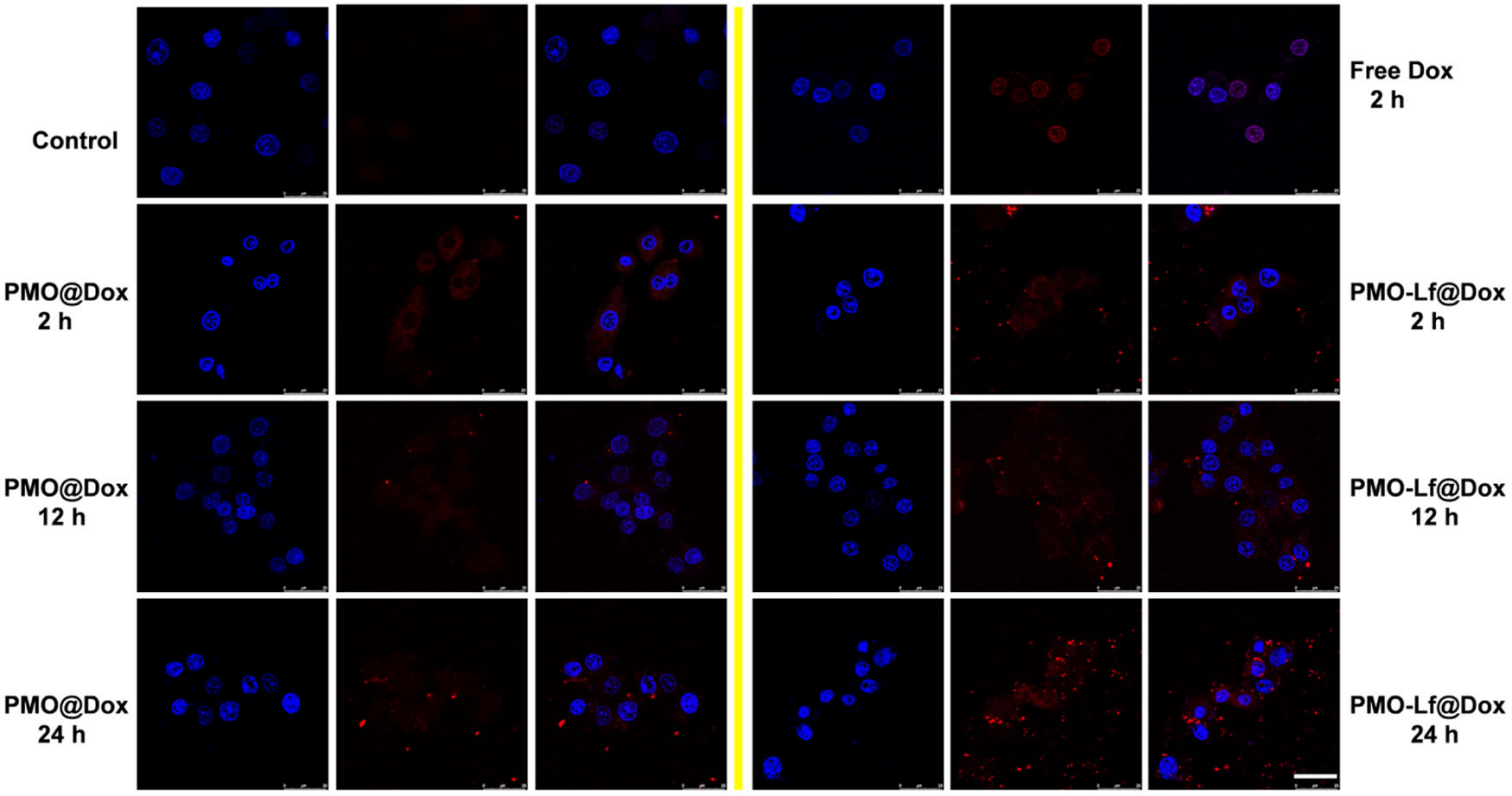
CLSM images of C6 cells incubated with free Dox, PMO@Dox and PMO-Lf@Dox for different times. The red and blue fluorescence comes from Dox and DAPI, respectively. Untreated cells are used as a control. Scale: 25 μm

Next, the cellular uptake of C6 cells after incubating free Dox, PMO@Dox and PMO-Lf@Dox were investigated. As shown in Fig. 4, after C6 cells incubating free Dox for only 2 h, the fluorescent of Dox is observed in the nucleus. After the corresponding incubation of PMO@Dox and PMO-Lf@Dox, only the red fluorescence of Dox can be seen in the cytoplasm. When the incubation time is extended, the fluorescence intensity in the cells increase, and the Dox fluorescence is gradually seen in the nucleus. When the incubation time is 24 h, both the PMO@Dox and PMO-Lf@Dox groups can see the red fluorescence of Dox in the nucleus, but the fluorescence intensity of the C6 cells treated by PMO-Lf@Dox is significantly higher than that of PMO @Dox.

**Fig. 5:**
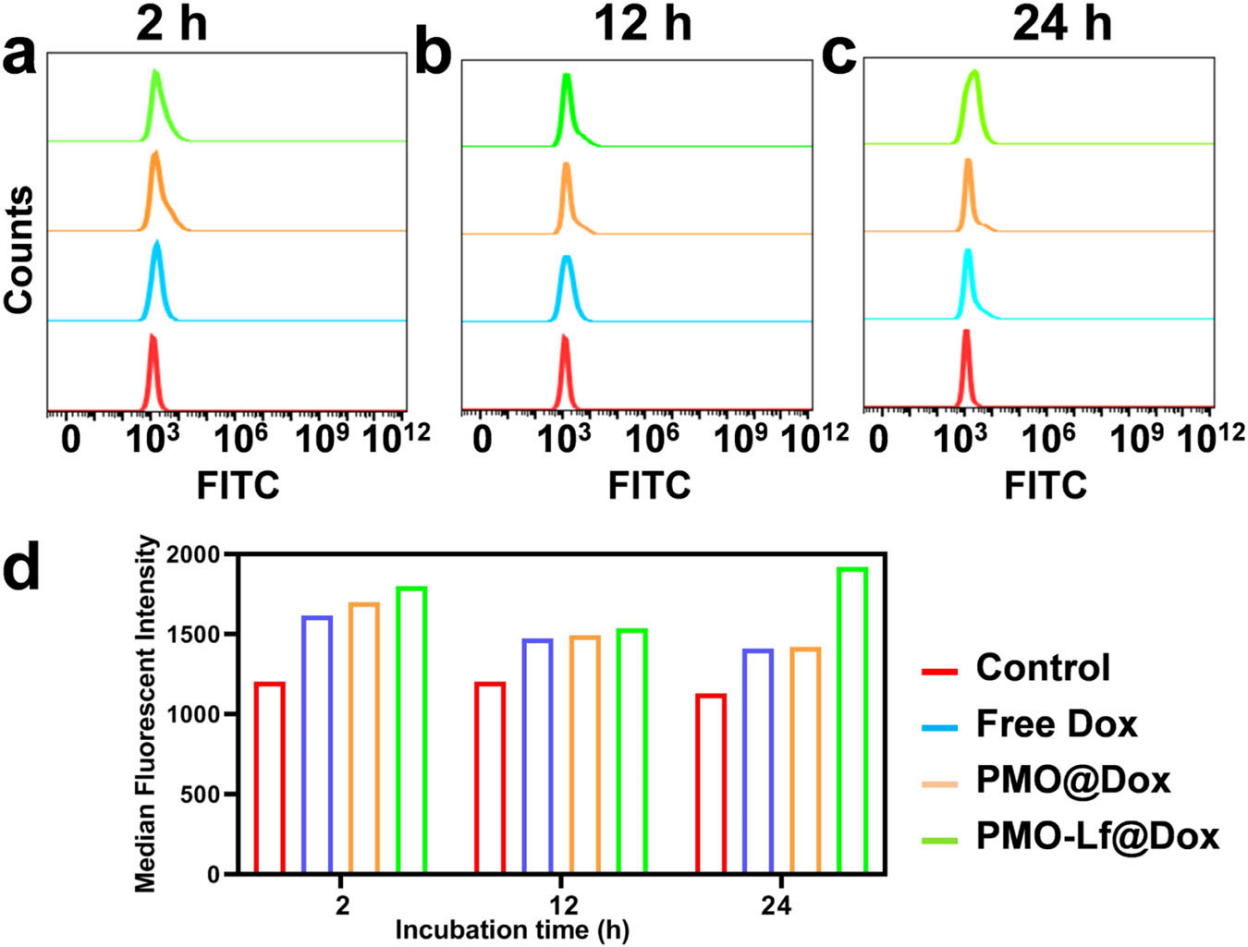
Flow cytometry analysis of the fluorescence intensity of C6 cells incubated with free Dox, PMO@Dox and PMO-Lf@Dox for (**a**) 2 h, (**b**) 12 h and (**c**) 24 h. **d** The corresponding quantitative results of fluorescence intensity. Untreated cells serve as a control group

The uptake of free Dox, PMO@Dox and PMO-Lf@Dox by C6 cells was quantitatively analyzed by flow cytometry. As shown in Fig. 5, when the incubation time is 2 h, the fluorescence intensity of free Dox, PMO@Dox and PMO-Lf@Dox gradually increased, indicating that more PMO-Lf@Dox enter C6 cells. The same trend is observed at 12 h of incubation. When the incubation time is further extended to 24 h, the uptake of PMO-Lf@Dox by C6 cells is significantly higher than that of PMO@Dox. The co-localization of DAPI and Dox was analyzed by using Image J and the results are shown in Supplementary Fig. S2. When the incubation time is 24 h, red fluorescence of Dox in the nucleus can be depicted both in the PMO@Dox and PMO-Lf@Dox groups. The fluorescence intensity of the C6 cells treated by PMO-Lf@Dox is significantly higher than that of PMO @Dox.

**Fig. 6:**
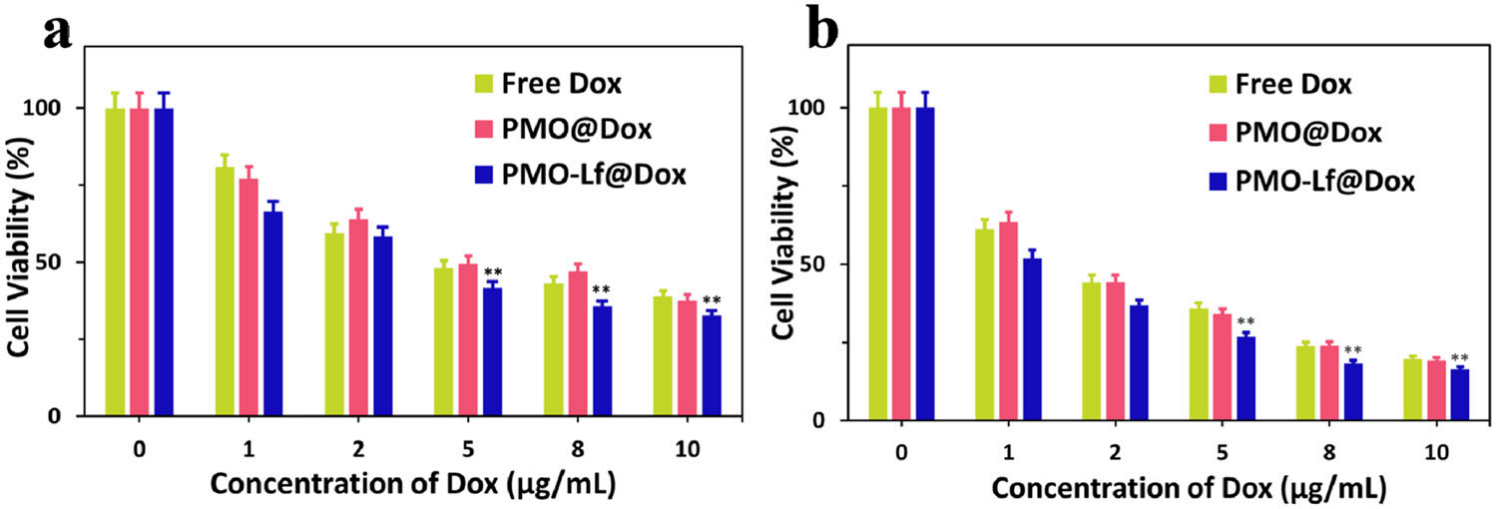
The survival rate of C6 cells incubated with free Dox, PMO@Dox and PMO-Lf@Dox for (**a**) 24 h and (**b**) 72 h. **p < 0.05 analyzed by One-way ANOVA

Finally, the therapeutic efficacy of PMO-Lf@Dox on C6 cells was studied. After free PMO, PMO-Lf, free Dox, PMO@Dox and PMO-Lf@Dox were incubated for C6 cells for 24 h, the cell inhibition rate results of each group are shown in Fig. 6a and Supplementary Fig. S3. For pure PMO and PMO-Lf, the cell viability of C6 cells remains as high as 90% even if the concentration of them reaches up to 10 μg/mL. After loading the chemotherapy drug Dox, the cell survival rates decrease to 38% and 33% in the PMO@Dox and PMO-Lf@Dox groups respectively, when the concentration of Dox is 10 μg/mL (Fig. 6a). A similar trend was observed when the incubation time was extended to 72 h (Fig. 6b).

## Discussion

In this study, we developed a biodegradable PMO modified with lactoferrin (Lf) ligands, which enabled the glioma-targeted drug delivery for cancer therapy. This drug delivery system was demonstrated to have large pore volume for high loading capacity of chemotherapeutic drugs Dox. Glutathione degradation experiments also revealed that the PMO nanoparticles can degraded in the tumor reducing microenvironment. This kind of nanoplatform has very little toxicity to cells and less hemolysis rate, which possess good biocompatibility and was beneficial to the drug delivery in blood vessel. In vivo analysis of the changes in heart, liver, spleen, lung and kidney and the mouse body weight further demonstrated the good cytocompatibility and biocompatibility of the nanoplatform. These features endowed the prepared PMO with excellent capability to be suitable as a carrier material for cancer drugs.

Currently, surgery resection is still the main treatment strategy for glioma. However, due to the highly invasive growth pattern of the tumor and the anatomical structure of central nerves system, tumor recurrence after initial resection is very common. Chemotherapy has been proposed as one of the adjuvant therapeutic options for recurrent tumors [25–27]. To improve the chemotherapy efficiency for glioma and reduce severe system toxicity to normal tissues, numerous nanoparticle-based drug delivery systems have been constructed in previous studies [28–31]. Periodic mesoporous organosilica nanoparticles have been widely used in cancer therapy by the virtue of high specific surface area, tunable mesoporous channels, and large pore volume properties. Furthermore, the surface of PMO can be easily modified with various organic groups to improve targeting-drug-delivery. However, traditional PMOs are difficult to degrade in vivo, which raised the concern about the safety of PMOs as nanomedicine carrier [32]. In this work, a reductive microenvironment-triggered biodegradation PMO of less than 100 nm in particles diameter fabricated by a facile and large-scale sol-gel strategy was employed for target delivery and concurrent reduction-responsive releasing of anticancer drug. Cell cytocompatibility experiments found that the cell viability was still maintained at 90% and intravenous injection of PMO for 1 week did not cause obvious changes in heart, liver, spleen, lung and kidney. Hemolysis experiments also showed the hemolysis rate was less than 2%. In vivo experiments and analysis of changes in mouse body weight all showed good cytocompatibility and biocompatibility of the material. Glutathione degradation experiments also showed that after culture in glutathione for 3 days, the edges of the PMO nanoparticles became blurred and finally almost completely degraded. No spherical morphology was observed, solving the problem of long-term accumulation of materials in vivo.

PMOs have been used as chemotherapy drugs vehicles for cancer treatment. Wu and his colleagues described a benzene-bridged PMO functionalized with high loadings of carboxylic acid groups for controlled loading and release of DOX in cancer therapy [33]. Qian and co-workers reported a PMO simultaneously encapsulate aloe-emodin and IR820. The resulting material allowed synergistic chemo-photothermal antitumor therapy [34]. However, few of the published papers focused on improving tumor targeting efficiency of the PMO based drug delivery system by conjunct lactoferrin (Lf). Lf is a multifunctional glycoprotein, which belongs to the transferrin family. Receptors of Lf has been well known to be over-expressed in glioma cells and brain endothelial cells. It also has the advantage of low expression in healthy tissues, so it does not compete with exogenous ligands. Because of their ability to target glioma cells and cross the blood-brain-barrier, Lf has been widely studied in targeting nanomedicine delivery [28, 30, 35, 36]. However, previous studies on Lf have mainly focused on assembling Lf itself into nanoparticles or combining it with liposomes, polymers, or Iron oxide nanoparticles for drug delivery [13–15]. To the best of our knowledge, Lf has not been modified on PMO vehicles in previous researches.

This study quantitatively analyzed the uptake and found that the uptake of PMO-Lf@Dox by C6 cells was higher than that of PMO@Dox. This indicates that lactoferrin modification can significantly increase the uptake of C6 cells, consistent with the conclusions of other studies. For example, Bin et al. evaluated the glioma targeting efficiency of three different ligands and found that Lf can significantly enhance tumor cell uptake [12]. Pang et al. found that lf-conjugated polymers significantly improve delivery efficiency [13]. In addition, in the study of the therapeutic effect of PMO-Lf@Dox on C6 cells, the results show that Lf-modified PMO enhanced the inhibition of Dox on C6 cells after 24 h of incubation, and the inhibitory effect of the modified group was better than that of the unmodified group after 72 h of incubation. Finally, in vivo experimental studies on therapeutic effects, we found that tumor-bearing control mice lost more weight than treated mice, but the treated mice were closer to normal healthy mice in growth, and the treated group inhibited tumor growth. The treated group, namely lf-modified PMO, had a better therapeutic effect and enhanced the inhibitory effect of Dox on C6 cells. This result is consistent with the conclusion of Janjua et al. that the experimental group modified with Lf can increase glioma cell apoptosis in vitro experiments.

Although the effectiveness of using DOX in treating glioma is limited by its inability to penetrate the BBB, it has been demonstrated in vitro that DOX can effectively induce glioma cell death and that DOX-coated nanoparticles can penetrate a model of BBB made up of a monolayer of Madin–Darby canine kidney transfected with multidrug resistant protein [29]. This study quantitatively analyzed the cellular uptake efficiency and found that the cellular uptake efficiency of PMO-Lf@Dox by C6 cells was higher than that of PMO@Dox. This indicates that lactoferrin modification can significantly increase the cell targeting ability to enhanced the C6 cells uptake of DOX. In addition, in the study of the therapeutic effect of PMO-Lf@Dox on C6 cells, the results show that Lf-modified PMO enhanced the inhibition of Dox on C6 cells after 24 h of incubation, and the inhibitory effect of the modified group was better than that of the unmodified group after 72 h of incubation. Finally, in vivo studies also demonstrated the treated group inhibited tumor growth effectively. The Lf-modified PMO group had a better therapeutic effect and enhanced the inhibitory effect of Dox on C6 cells.

The current work has the following limitations. First, in vivo tumor treatment experiments have not been carried out. This study requires the construction of an orthotopic model of glioblastoma and testing the targeting of lactoferrin. However, the experiment was limited by the existence of the blood-brain barrier. In future research, we will conduct in-depth research in this area.

In conclusion, thioether groups incorporated PMO is successfully synthesized with a uniform size of about 99 nm and excellent dispersibility in water and has high surface area and pore volume. The obtained PMO are biodegradable and has Dox loading capacity of about 20%. The cellular uptake of PMO-Lf@Dox by C6 Rat Glioma Cell increases compared with PMO@Dox. The therapeutic efficacy results show that Lf-modified PMO enhances the inhibitory effect of Dox on C6 cells when incubated for 24 h and 72 h. This nanoplatform, with its ability to specifically target gliomas, high loading efficiency for the drug doxorubicin, and biodegradability in the tumor microenvironment, shows great promise in improving cancer treatment for glioma.

### Supplementary information

Supplemenaty Information

## References

[1] Gusyatiner O, Hegi ME (2018). Glioma epigenetics: from subclassification to novel treatment options. Semin Cancer Biol.

[2] Sarkaria JN, Hu LS, Parney IF, Pafundi DH, Brinkmann DH, Laack NN (2018). Is the blood-brain barrier really disrupted in all glioblastomas? A critical assessment of existing clinical data. Neuro Oncol.

[3] Wang Z, Sun H, Yakisich JS (2014). Overcoming the blood-brain barrier for chemotherapy: limitations, challenges and rising problems. Anticancer Agents Med Chem.

[4] Tang W, Fan W, Lau J, Deng L, Shen Z, Chen X (2019). Emerging blood-brain-barrier-crossing nanotechnology for brain cancer theranostics. Chem Soc Rev.

[5] Ji X, Wang H, Chen Y, Zhou J, Liu Y (2019). Recombinant expressing angiopep-2 fused anti-VEGF single chain Fab (scFab) could cross blood-brain barrier and target glioma. AMB Express.

[6] Kou L, Hou Y, Yao Q, Guo W, Wang G, Wang M (2018). L-Carnitine-conjugated nanoparticles to promote permeation across blood-brain barrier and to target glioma cells for drug delivery via the novel organic cation/carnitine transporter OCTN2. Artif Cells Nanomed Biotechnol.

[7] Presti S, Manti S, Parisi GF, Papale M, Barbagallo IA, Li Volti G, et al. Lactoferrin: cytokine modulation and application in clinical practice. J Clin Med. 2021;10:5482. 10.3390/jcm10235482 PMC8658270 34884183

[8] Mokhtar S, Khattab SN, Elkhodairy KA, Teleb M, Bekhit AA, Elzoghby AO (2022). Methotrexate-lactoferrin targeted exemestane cubosomes for synergistic breast cancer therapy. Front Chem.

[9] Sabra S, Agwa MM (2020). Lactoferrin, a unique molecule with diverse therapeutical and nanotechnological applications. Int J Biol Macromol.

[10] Cutone A, Rosa L, Ianiro G, Lepanto MS, Bonaccorsi di Patti MC, Valenti P, et al. (2020) Lactoferrin’s anti-cancer properties: safety, selectivity, and wide range of action. Biomolecules. 2020;10:456. 10.3390/biom10030456 PMC7175311 32183434

[11] Tomitaka A, Arami H, Gandhi S, Krishnan KM (2015). Lactoferrin conjugated iron oxide nanoparticles for targeting brain glioma cells in magnetic particle imaging. Nanoscale.

[12] Ji B, Maeda J, Higuchi M, Inoue K, Akita H, Harashima H (2006). Pharmacokinetics and brain uptake of lactoferrin in rats. Life Sci.

[13] Pang Z, Feng L, Hua R, Chen J, Gao H, Pan S (2010). Lactoferrin-conjugated biodegradable polymersome holding doxorubicin and tetrandrine for chemotherapy of glioma rats. Mol Pharmaceutics.

[14] Janjua TI, Cao Y, Ahmed-Cox A, Raza A, Moniruzzaman M, Akhter DT (2023). Efficient delivery of Temozolomide using ultrasmall large-pore silica nanoparticles for glioblastoma. J Control Rel.

[15] Qi N, Zhang S, Zhou X, Duan W, Gao D, Feng J (2021). Combined integrin alpha(v)beta(3) and lactoferrin receptor targeted docetaxel liposomes enhance the brain targeting effect and anti-glioma effect. J Nanobiotechnol.

[16] Liu S, Zhang SM, Ju RJ, Xiao Y, Wang X, Song XL (2017). Antitumor efficacy of Lf modified daunorubicin plus honokiol liposomes in treatment of brain glioma. Eur J Pharm Sci.

[17] Kuo YC, Cheng SJ (2016). Brain targeted delivery of carmustine using solid lipid nanoparticles modified with tamoxifen and lactoferrin for antitumor proliferation. Int J Pharm.

[18] Li H, Tong Y, Bai L, Ye L, Zhong L, Duan X (2018). Lactoferrin functionalized PEG-PLGA nanoparticles of shikonin for brain targeting therapy of glioma. Int J Biol Macromol.

[19] Rahmani S, Mauriello Jimenez C, Aggad D, Gonzalez-Mancebo D, Ocana M, Lamiaa MAA, et al. Encapsulation of upconversion nanoparticles in periodic mesoporous organosilicas. Molecules. 2019;24:4054. 10.3390/molecules24224054 PMC6891486 31717490

[20] Teng Z, Li W, Tang Y, Elzatahry A, Lu G, Zhao D (2019). Mesoporous organosilica hollow nanoparticles: synthesis and applications. Adv Mater.

[21] Attia MF, Swasy MI, Ateia M, Alexis F, Whitehead DC (2020). Periodic mesoporous organosilica nanomaterials for rapid capture of VOCs. Chem Commun.

[22] Arcella A, Oliva MA, Staffieri S, Aalberti S, Grillea G, Madonna M (2015). In vitro and in vivo effect of human lactoferrin on glioblastoma growth. J Neurosurg.

[23] Peng X, Chen K, Liu W, Cao X, Wang M, Tao J (2020). Soft mesoporous organosilica nanoplatforms improve blood circulation, tumor accumulation/penetration, and photodynamic efficacy. Nanomicro Lett.

[24] Teng Z, Su X, Lee B, Huang C, Liu Y, Wang S (2014). Yolk–shell structured mesoporous nanoparticles with thioether-bridged organosilica frameworks. Chem Mater.

[25] Ducray F (2011). Chemotherapy for diffuse low-grade gliomas in adults. Rev Neurol.

[26] Jacobo JA, Mejia-Perez S, Moreno-Jimenez S (2021). The role of neoadjuvant therapy to improve the extent of resection in “unresectable” gliomas. World Neurosurg.

[27] Taal W, Bromberg JE, van den Bent MJ (2015). Chemotherapy in glioma. CNS Oncol.

[28] Patel D, Wairkar S, Yergeri MC (2020). Current developments in targeted drug delivery systems for glioma. Curr Pharm Des.

[29] Norouzi M, Yathindranath V, Thliveris JA, Kopec BM, Siahaan TJ, Miller DW (2020). Doxorubicin-loaded iron oxide nanoparticles for glioblastoma therapy: a combinational approach for enhanced delivery of nanoparticles. Sci Rep.

[30] Luiz MT, Delello Di Filippo L, Tofani LB, de Araújo JTC, Dutra JAP, Marchetti JM (2021). Highlights in targeted nanoparticles as a delivery strategy for glioma treatment. Int J Pharm.

[31] Lu G, Wang X, Li F, Wang S, Zhao J, Wang J (2022). Engineered biomimetic nanoparticles achieve targeted delivery and efficient metabolism-based synergistic therapy against glioblastoma. Nat Commun.

[32] Park S, Santha Moorthy M, Ha CS (2014). Periodic mesoporous organosilicas for advanced applications. NPG Asia Mater.

[33] Wu HY, Shieh FK, Kao HM, Chen YW, Deka JR, Liao SH (2013). Synthesis, bifunctionalization, and remarkable adsorption performance of benzene-bridged periodic mesoporous organosilicas functionalized with high loadings of carboxylic acids. Chemistry.

[34] Qian C, Wang M, Wang L, Wang H, Li Y, Tang X (2022). Functionalized periodic mesoporous organosilica nanoparticles for combinational chemo-photothermal antitumor therapy. ACS Appl Nano Mater.

[35] Kumari S, Ahsan SM, Kumar JM, Kondapi AK, Rao NM (2017). Overcoming blood brain barrier with a dual purpose Temozolomide loaded Lactoferrin nanoparticles for combating glioma (SERP-17-12433). Sci Rep.

[36] Marei HE (2022). Multimodal targeting of glioma with functionalized nanoparticles. Cancer Cell Int.

