# Supplementary material for: Enhanced anti-glioma efficacy of biodegradable periodic mesoporous organosilica nanoparticles through target delivery of chemotherapeutics
Source: J Mater Sci Mater Med. 2023 Oct 4;34(10):48. doi: 10.1007/s10856-023-06747-x (PMC10550876; doi:10.1007/s10856-023-06747-x)
Supplement: Supplementary file 1 — Supplemenaty Information [file 10856_2023_6747_MOESM1_ESM.doc]

**Supporting Information**

Enhanced anti-glioma efficacy of biodegradable periodic mesoporous organosilica nanoparticles through target delivery of chemotherapeutics

Min Dong^1#^, Ying Liu^2#^, Biao Liu^1^, Jin Peng^3^, Yuxia Tang^4^, Guangming Lu^5*^, Haibin Shi^6*^, Feipeng Zhu4[[1]](#footnote-2)*

1 Department of Comparative Medicine, Jinling Hospital, School of Medicine, Nanjing University, 305 East Zhongshan Road, Nanjing 210002, P.R. China.

2 School of Intelligent Manufacturing and Electronic Engineering, Wenzhou University of Technology, Wenzhou 325025, P.R.China.

3 Intervention Department, Chenggong Hospital Affiliated to Xiamen University, 94-96 Wenyuan Road, Xiamen 361003, P.R.China.

4 Department of Radiology, The First Affiliated Hospital of Nanjing Medical University, 300 Guangzhou Road, Nanjing 210029, P.R. China.

5 Department of Medical Imaging, Jinling Hospital, School of Medicine, Nanjing University, 305 East Zhongshan Road, Nanjing 210002, P.R. China.

6 Department of Interventional Radiology, The First Affiliated Hospital of Nanjing Medical University, 300 Guangzhou Road, Nanjing 210029, P.R. China.

**Table S1. The pathologic changes of the different organs.**

| **Item** | | **Control** | **PMO** |
| --- | --- | --- | --- |
| **Heart** | Inflammatory cells | 0 | 0 |
|  | Degeneration | 0 | 0 |
|  | Necrosis | 0 | 0 |
| **Kidney** | Bruising | 1 | 2 |
|  | Degeneration | 0 | 0 |
|  | Inflammatory cells | 0 | 0 |
| **Liver** | Hepatocellular oedema | 2 | 1 |
|  | Inflammatory cells | 1 | 0 |
|  | Necrosis | 0 | 0 |
| **Lung** | Alveolar wall thickening | 0 | 1 |
|  | Inflammatory cells | 0 | 1 |
|  | Necrosis | 0 | 0 |
| **Spleen** | Extramedullary haematopoietic cells | 2 | 2 |
|  | Bruising | 1 | 0 |
|  | Necrosis | 0 | 0 |


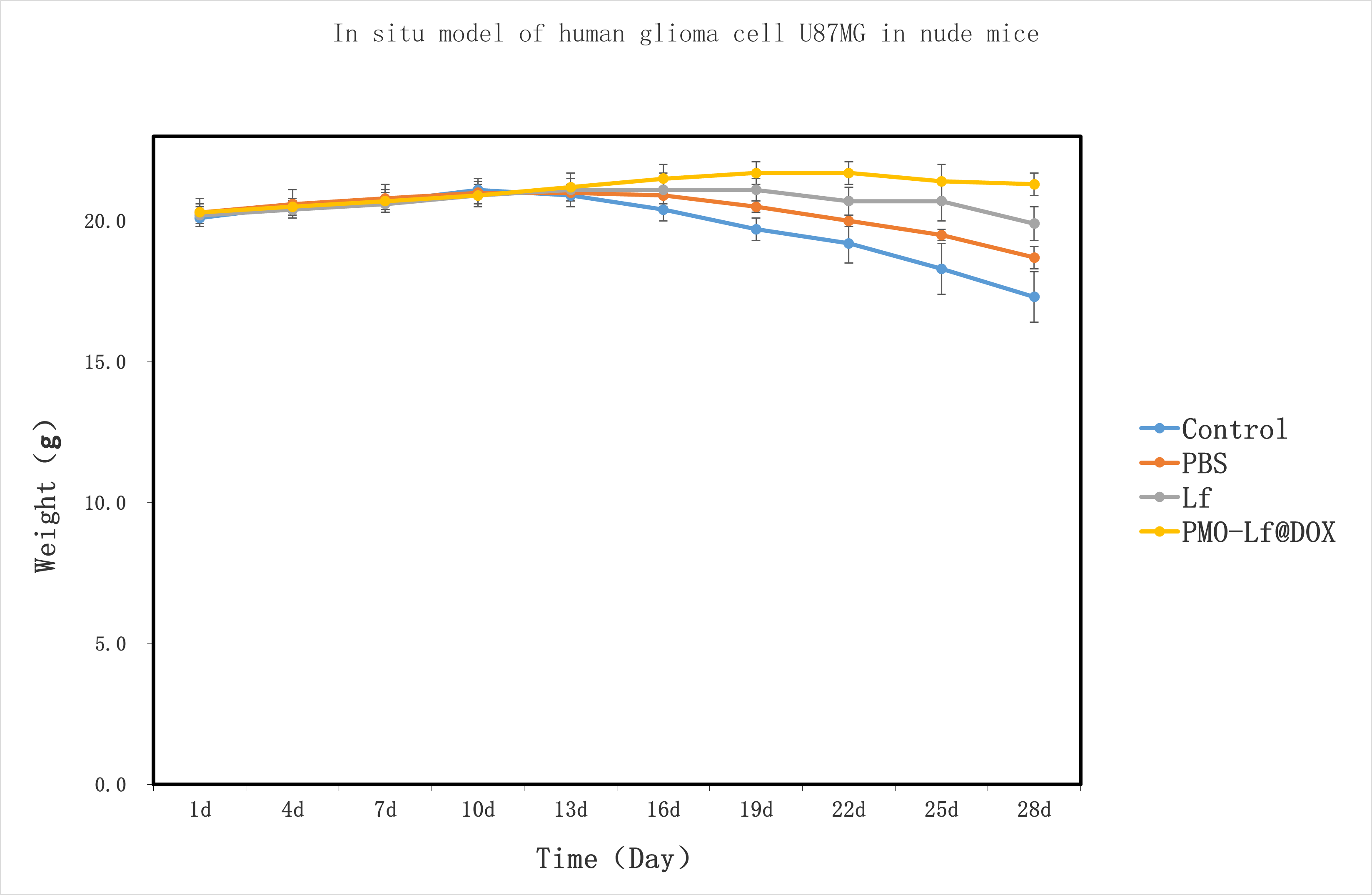


Figure S1. The body weight of tumor-bearing mice with different treatments.


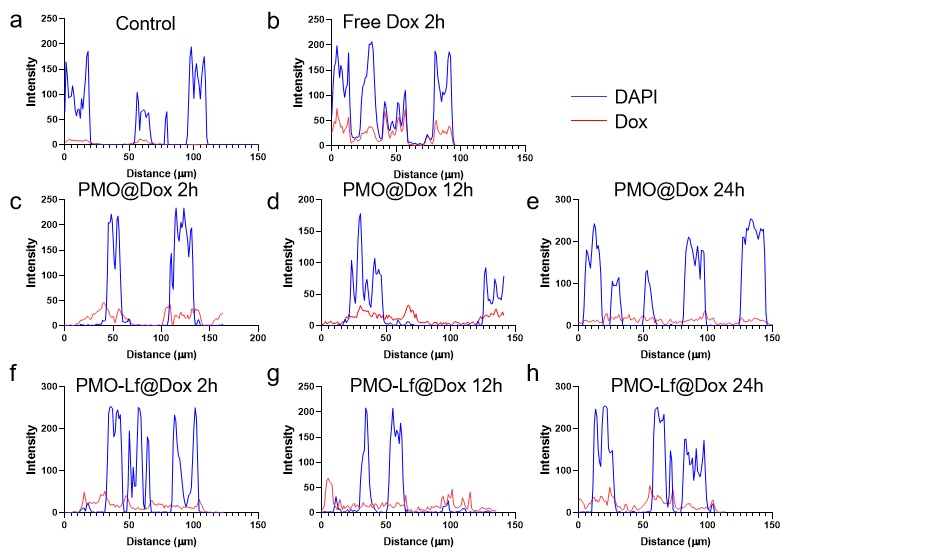


Figure S2. The co-localization of CLSM images of C6 cells incubated with free Dox, PMO@Dox and PMO-Lf@Dox for different times. The red and blue line is Dox and DAPI, respectively. Untreated cells are used as a control.


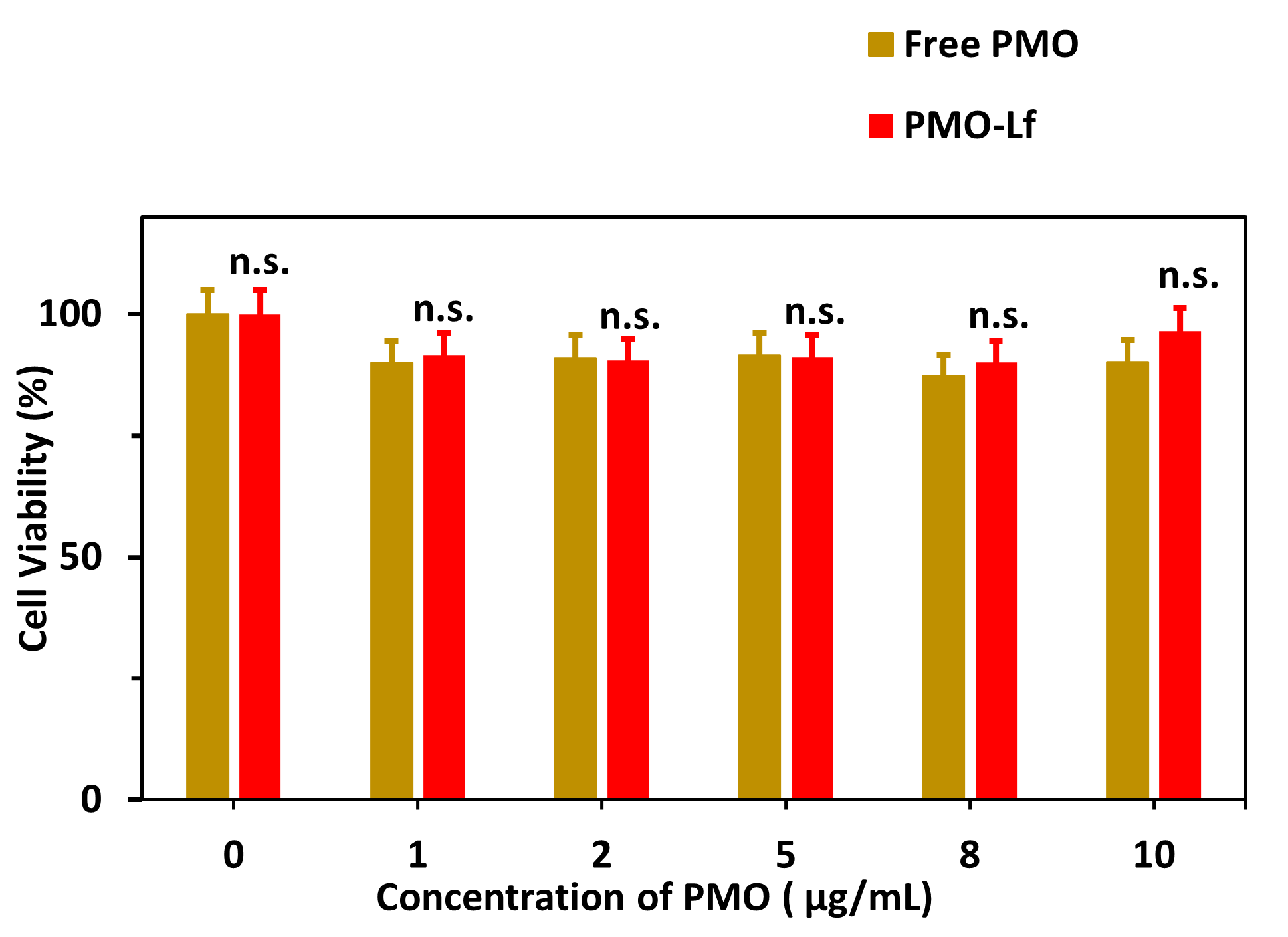


Figure S3. The cell survival rate of c6 cells incubated with PMO and PMO-Lf at different concentrations for 24h.

1. ^*^ Corresponding authors.

   Email address: zhufeipeng2008@163.com (F. Zhu), [shihb@vip.sina.com](mailto:shihb@vip.sina.com) (H. Shi), cjr.luguangming@vip.163.com (G. Lu).

   #These authors contributed equally to this work. [↑](#footnote-ref-2)
